# Evaluating a framework for tuberculosis screening among healthcare workers in clinical settings, Inner Mongolia, China

**DOI:** 10.1186/s12995-018-0192-y

**Published:** 2018-03-20

**Authors:** Shiming Cheng, Deanna Tollefson, Guangxue He, Yuan Li, Hui Guo, Shua Chai, Fangfang Gao, Fei Gao, Guoxin Han, Liping Ren, Yulin Ren, Jianbo Li, Lixia Wang, Jay K. Varma, Dongmei Hu, Haiying Fan, Fei Zhao, Emily Bloss, Yu Wang, Carol Y. Rao

**Affiliations:** 1 China Center for Disease Control and Prevention, 155 Changbai Road Changping District, Beijing, 102206 People’s Republic of China; 2 U.S. Centers for Disease Control and Prevention (CDC), 1600 Clifton Road NE, MS-93, Atlanta, GA 30329 USA; 3 U.S. Centers for Disease Control and Prevention (CDC), Beijing, China; 4 Inner Mongolia Center for Tuberculosis Control and Prevention, Middle Hugao Rd, New District, Hohhot, Inner Mongolia 010080 People’s Republic of China; 5 Ulanqab General Hospital, No.157, Jiefang Road, Jining District, Ulanqab, Inner Mongolia 012000 People’s Republic of China

**Keywords:** Case detection, Medical surveillance, Clinically diagnosed cases, Active case finding, Healthcare workers, X-ray, Tuberculosis, Tuberculin skin test

## Abstract

**Background:**

Health care workers are at high risk for tuberculosis (TB). China, a high burden TB country, has no policy on medical surveillance for TB among healthcare workers. In this paper, we evaluate whether China’s national TB diagnostic guidelines could be used as a framework to screen healthcare workers for pulmonary TB disease in a clinical setting in China.

**Methods:**

Between April–August 2010, healthcare workers from 28 facilities in Inner Mongolia Autonomous Region, China were eligible for TB screening, comprised of symptom check, chest X-ray and tuberculin skin testing. Healthcare workers were categorized as having presumptive, confirmed, or clinically-diagnosed pulmonary TB, using Chinese national guidelines.

**Results:**

All healthcare workers (N=4347) were eligible for TB screening, of which 4285 (99%) participated in at least one TB screening test. Of the healthcare workers screened, 2% had cough for ≥ 14 days, 3% had a chest X-ray consistent with TB, and 10% had a tuberculin skin test induration ≥ 20 mm. Of these, 124 healthcare workers were identified with presumptive TB (i.e., cough for ≥ 14 days in the past 4 weeks or x-ray consistent with TB). Twelve healthcare workers met the case definition for clinically-diagnosed pulmonary TB, but none were diagnosed with TB during the study period.

**Conclusion:**

A substantial proportion of healthcare workers in Inner Mongolia had signs, symptoms, or test results suggestive of TB disease that could have been identified using national TB diagnostic guidelines as a screening framework. However, achieving medical surveillance in China will require a framework that increases the ease, accuracy, and acceptance of TB screening in the medical community. Routine screening with improved diagnostics should be considered to detect tuberculosis disease among healthcare workers and reduce transmission in health care settings in China.

## Background

Since 1990, China has experienced substantial declines in the burden of tuberculosis (TB) nationally [1], yet the country continues to have the third largest number of incident TB cases and the highest number of multidrug resistant (MDR) TB cases in the world [2]. Healthcare workers (HCWs) are a high risk group for TB infection in China [3–8], possibly due to inadequate infection control practices in healthcare settings [3]. However, medical surveillance of HCWs for TB disease is not routine, and there are no guidelines to screen HCWs for TB disease in China [9]. As such, HCWs may develop TB disease, not be diagnosed in a timely manner and thus contribute to ongoing TB transmission. Accurate and timely diagnosis of TB disease is essential to ensure proper patient care and appropriate public health response.

Guidelines for routine TB screening among HCWs can help reduce ongoing transmission of TB and be used to monitor and evaluate existing nosocomial TB infection control policies [9]. In lieu of these, China’s national TB diagnostic guidelines can serve as a framework for routine screening of HCWs for TB disease [10, 11], but the application of the diagnostic guidelines in this context has not been evaluated. To address this gap, we evaluated using China’s national TB diagnostic guidelines to screen HCWs for pulmonary TB disease in a clinical setting in Inner Mongolia. Inner Mongolia is an Autonomous Region in northern China, with a population of approximately 24 million people in the 2010 census. In 2010, the notification rate of active TB in China’s Inner Mongolia Autonomous Region was 74.1 per 100,000 and the notification rate for smear positive TB was 35.4 per 100,000 [12].

## Methods

### Study population

Facilities that diagnosed TB cases were selected based on their capability to conduct chest X-rays and their willingness to participate. All HCWs in the 28 selected healthcare facilities were eligible to participate. A HCW was defined as any person who received payment for working in the facility, regardless of the type of employment (e.g., clinicians, nurses, laboratorians, and administrative, janitorial, and cafeteria staff). The number of eligible HCWs at a facility ranged from 9 to 1000. HCWs undergoing TB treatment were excluded.

### Definitions

We used 2008 Chinese national diagnostic guidelines to define a TB case. Chinese national guidelines rely on clinical judgement based on symptoms, chest radiography, tuberculin skin test (TST), and smear microscopy to diagnose TB; the national guidelines do not include use of culture for TB diagnosis. Per these guidelines, a confirmed pulmonary TB case was defined as a person with sputum smear positive acid-fast bacilli (AFB) results; a clinically-diagnosed TB case was defined as a person with a chest X-ray consistent with TB and either a cough for more than 14 days in the past 4 weeks or a tuberculin skin test reading ≥20 mm [10, 11]. We defined a presumptive TB case as a person who, per Chinese national guidelines, was eligible for smear microscopy testing: i.e., one who had a chest X-ray consistent with TB or one who had cough for more than 14 days in the past 4 weeks.

### Screening for TB

Between April and August 2010, HCWs were screened for active pulmonary TB disease following Chinese national guidelines, the first step of which included offering all HCWs a symptom screen, a chest x-ray, and TST. Project personnel administered a standardized questionnaire that included a TB symptom screen, such as cough for more than 14 days, and collected information on HCW demographics and risk factors for TB disease. A sub-set of participating health care workers were offered chest X-rays and TSTs; these HCWs were conveniently sampled by facility staff. Radiologists from each participating clinic independently reviewed chest X-rays for participating HCWs from their own facilities; no additional training was provided on taking and reading chest X-rays. Study staff were trained by China CDC to place TSTs and read and interpret the results, since TSTs were not routinely administered. Study staff performed a single-step TST using 5 IU (0.1 ml) of domestically-produced tuberculin derived from *M. bovis* BCG (Chengdu Institute of Biological Products, Chengdu, China).

Per national guidelines, facility staff were to refer an HCW for sputum smear microscopy if he/she had a chest X-ray suggestive of TB or symptoms of cough greater than 2 weeks, and were to follow routine facility procedures to record and report diagnosed cases. Presumptive TB cases should provide spot, morning and night sputum specimens. To identify TB cases, we searched the national electronic TB surveillance system for HCWs who were eligible for sputum smear evaluation (matching by name, age, gender and current address) to ascertain if they were diagnosed with active TB within 5 years of the project (from July 2010–July 2015).

### Data analysis

All data were collected on paper forms and double-entered into Microsoft Excel (Redmond, WA, USA). Project staff conducted periodic site visits to monitor data quality and to provide ongoing training, as necessary. Frequencies and proportions were calculated and compared for all HCWs at each stage of the TB screening process. Data were analyzed using SAS 9.3 (SAS Institute, Cary, NC, USA).

### Ethical considerations

HCWs provided written informed consent. The Chinese Ethical Committee for Tuberculosis Operational Research and the Chinese Center for Disease Control and Prevention approved this project.

## Results

**Fig. 1:**
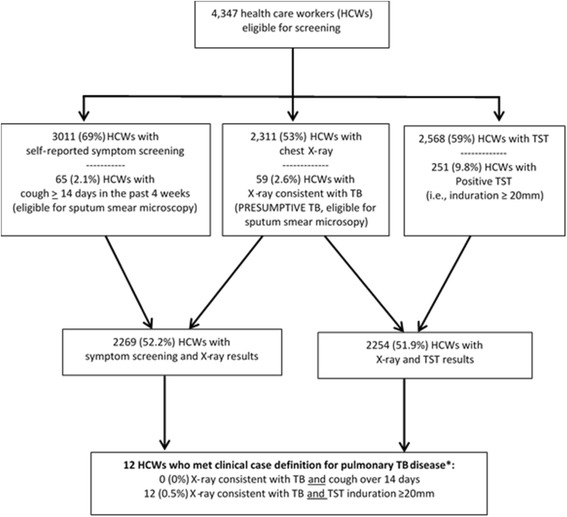
TB screening and diagnostic algorithm as used among health care workers (HCWs) in Inner Mongolia. *No HCWs were actually diagnosed with active pulmonary TB during the course of the evaluation.

Within the 28 facilities, 4347 HCWs were eligible to participate in the study. Of these, 3011 (69.3%) underwent symptom screening, 2311 (53%) received a chest x-ray, and 2568 (59%) received a TST (Fig. 1); 4285 HCWs participated in at least one of these screenings. There were no significant differences in the demographic characteristics between HCWs participating in each of the screening methods (data not shown). Among HCWs screened for symptoms, most were primarily medical doctors or nurses (47%), females (66%), never smokers (77%), and at least 40 years old (53%); half of participants had spent more than 15 years working in health care settings (52%) (Table 1). Most HCWs had never had a TST (80%) and, of the 591 HCWs who ever had a TST, 217 (37%) had a skin test within the last 5 years.

**Table 1:** Characteristics of health care workers (HCWs) screened for symptoms or sputum testing or clinically-diagnosed TB

Characteristic	HCWs with symptom screening (*n* = 3011^a^)	HCWs eligible for sputum testing^b^ (*n* = 124^a^)	Met case definition for clinically-diagnosed TB^c^ (n = 12)
	n (%)	n (%)	n (%)
DEMOGRAPHICS			
Sex			
Female	1997 (66%)	67 (54%)	9 (75%)
Male	1014 (34%)	57 (46%)	3 (25%)
Age, years			
18–29	598 (20%)	14 (11%)	1 (8%)
30–39	824 (27%)	32 (26%)	4 (33%)
40–49	1029 (34%)	40 (33%)	4 (33%)
≥ 50	558 (19%)	36 (30%)	3 (25%)
Education			
College or above	852 (28%)	52 (43%)	6 (50%)
Some college or tech school	1480 (49%)	48 (39%)	4 (33%)
High school or below	674 (22%)	22 (18%)	2 (17%)
Self-reported BCG			
Did not have BCG	1521 (51%)	55 (45%)	2 (17%)
Had BCG	1468 (49%)	66 (55%)	10 (83%)
Smoking status			
Never smoker	2319 (77%)	80 (66%)	10 (83%)
Former/current smoker	681 (23%)	41 (34%)	2 (17%)
TB infection and disease history			
Previous diagnosis of TB	147 (5%)	27 (22%)	4 (33%)
Previous TST	591 (20%)	37 (30%)	7 (58%)
Tested within last 5 years	217 (37%)	10 (27%)	2 (29%)
Previously positive TST^d^	239 (41%)	24 (67%)	4 (57%)
WORK-RELATED TB RISK FACTORS			
Work type			
Clinician or nurse	1420 (47%)	62 (51%)	7 (58%)
Clinical support^f^	713 (24%)	25 (20%)	1 (8%)
Administrative	411 (14%)	21 (17%)	2 (17%)
Other (e.g., cleaner, cook)	462 (15%)	14 (11%)	2 (17%)
Working time in health care			
≤ 5 years	746 (25%)	23 (19%)	2 (18%)
6–15 years	679 (23%)	19 (16%)	4 (36%)
> 15 years	1530 (52%)	78 (65%)	5 (45%)
Direct contact with TB cases			
TB patients	1295 (43%)	83 (68%)	10 (83%)
Coworkers with history of TB	254 (8%)	19 (16%)	4 (33%)
Household member with TB	230 (8%)	10 (8%)	1 (8%)
Average daily time with patients			
0 h	803 (27%)	23 (19%)	1 (8%)
< 1 h	458 (15%)	21 (17%)	4 (33%)
1–4 h	506 (17%)	22 (18%)	3 (25%)
> 4 h	1234 (41%)	55 (45%)	4 (33%)
OTHER SCREENING RESULTS			
Symptoms in the last 4 weeks			
None	2714 (90%)	49 (40%)	7 (58%)
Cough only	191 (6%)	53 (43%)	3 (25%)
Fever or night sweats, no cough	35 (1%)	1 (0.8%)	1 (8%)
Fever or night sweats, any cough	31 (1%)	13 (10%)	0
TST (mm)	n=2568^e^		
< 5	714 (28%)	20 (18%)	0
5–14	1232 (48%)	49 (45%)	0
15–19	356 (14%)	24 (22%)	0
≥ 20	251 (10%)	15 (15%)	12 (100%)
X-ray results	n=2269^e^	n=103^e^	
Consistent with TB	57 (2%)	59 (57%)	12 (100%)
Abnormal, not consistent with TB	65 (3%)	1 (1%)	0
Normal	2147 (95%)	43 (42%)	0

Footnotes:

^a^Categories may not total n due to missing data

^b^ Defined as HCWs who had a chest X-ray consistent with TB OR a cough lasting ≥ 14 days in the past 4 weeks

^c^ Defined as HCWs who had a chest X-ray consistent with TB AND a TST induration ≥ 20 mm

^d^ HCW self-report of TST positivity. We did not ask what induration size was considered positive. Chinese guidelines categorize 5–9 mm as general positive, 10–19 mm as medium positive and ≥ 20 mm as very positive (See: *Ministry of Health People’s Republic of China. Diagnostic criteria for pulmonary tuberculosis. 2008*. [11])

^e^ The denominators in these sub-sections are a subset of the HCWs within the column. The denominators are: a subset of HCWs with symptom screening who received TST, a subset of HCWs with symptom screening who received chest x-ray, and a subset of HCWs eligible for sputum testing who received chest x-ray

^f^ Clinical support jobs were radiographer/X-ray technician, laboratorian, public health/preventive medicine, and pharmacists

Among HCWs screened for symptoms, the majority of HCWs reported no TB symptoms (2714, 90%) (Table 1). Among HCWs receiving TST, 251 (10%) had a TST ≥20 mm. Among HCWs receiving chest X-rays, 125 (5%) had an abnormal result; this included 59 (47%) chest X-rays that were consistent with TB.

X-ray and symptom screening identified 124 HCWs with presumptive TB, who were thus eligible for sputum smear evaluation: 59 who had X-rays consistent with TB and 65 who reported cough for more than 14 days in the past 4 weeks (Fig. 1). There were no cases with both X-ray consistent with TB and cough for more than 14 days in the past 4 weeks. An unknown number of HCWs who should have been referred for sputum smear microscopy completed the test during the study period (i.e., April–August 2010) as facility staff only reported positive smear tests for HCWs and no smear results were reported. Between July 2010 and July 2015, however, 1 out of the 124 HCW eligible for sputum smear was reported in the national electronic TB surveillance system as having active TB disease.

Of the 2269 participants who completed both the symptom screening and a chest x-ray, 12 HCWs (0.53% or 529 per 100,000; 95% confidence interval [208, 849]) met the case definition for clinically-diagnosed, active pulmonary TB (i.e., X-ray consistent with TB and TST induration of at least 20 mm). However, despite meeting the case definition for active pulmonary TB disease, these 12 cases were not diagnosed with TB by their healthcare facilities. The majority of HCWs meeting the case definition for clinically-diagnosed TB were clinical staff (i.e., medical doctors or nurses) (7/12), had direct contact with TB patients (10/12) and had been working in healthcare settings for at least 6 years (9/12) (Table 1). Two-thirds of the HCWs who met the case definition for clinically diagnosed pulmonary TB were asymptomatic (i.e. reported no fever, night sweats, or cough in the last 4 weeks) (7/11) while nearly three quarters of HCWs with X-rays consistent with TB were asymptomatic (43/59).

## Discussion

TB is an under-recognized disease among healthcare workers in China. This study found that in Inner Mongolia, using China’s national TB diagnostic guidelines as a framework to screen HCWs for TB could have identified 12 HCWs with TB. Twelve HCWs met national guideline definitions for clinically diagnosed TB, which is equivalent to a prevalence of 529 (95% CI 208, 849) TB cases per 100,000, a number higher than the national TB prevalence (108 per 100,000) [13] and the provincial TB prevalence for Inner Mongolia (74 per 100,000) [12]. However, during the study period, none of the HCWs meeting the criteria for clinically diagnosed TB were confirmed microbiologically, formally diagnosed with TB disease, or treated with anti-TB medications, and thus were not reported as TB cases. This suggests that while screening for TB among HCWs in China is urgently needed, using the national TB diagnostic algorithm as a framework for medical surveillance in itself may be insufficient. Effectiveness of screening programs may be hampered by lack of recognition of TB disease among HCWs, poor understanding or acceptance of diagnostic guidelines, and poor quality of tests used to diagnose TB, which would need to be addressed to achieve optimal medical TB surveillance in China.

Medical surveillance for occupationally-acquired diseases among HCWs is not routine in China [9] despite global recommendations to conduct on-site surveillance of TB among HCWs [14]. We found 20 % of participating HCWs reported ever having a TST and fewer than half of those had the test within the last 5 years. If an HCW is diagnosed with TB in China, it is not generally acknowledged as an occupationally-acquired infection [9], even though our study found a high proportion of HCWs who met the case definition for sputum referral or for clinically-diagnosed TB had work related exposures (i.e., contact with TB patients or with coworkers with TB). The participation rate for the initial screening was high, with 98.5% participating in at least one screening method, indicating that there is capacity to conduct medical surveillance if prioritized by hospital management. Regular TB screening of HCWs would facilitate early identification of TB in this vulnerable population and reduce risk of TB transmission in healthcare settings.

This evaluation identified challenges in uptake of the current national TB diagnostic algorithm as a framework to screen HCWs for TB. Namely, the study found it difficult to achieve HCW participation in the full screening process, especially with smear microscopy. The low yield of smear positive TB cases identified in this study suggests that HCWs who should have been referred for sputum smear testing may not have completed testing. Previous studies in China have found that persons eligible for sputum smears are frequently not offered the test, and the number of individuals who complete a sputum exam among those offered a test is even lower [15, 16]. Most of the presumptive TB cases and participants who met the case definition of clinically-diagnosed pulmonary TB in this evaluation were asymptomatic which could have affected the perceived urgency for sputum testing. However, other barriers to being offered and completing sputum exams in China have been found to include economic constraints and innate beliefs held by both doctors and presumptive TB cases that sputum tests are unimportant [15, 16]. In addition, China does not have established protocols for referral and follow-up of asymptomatic HCWs with suspected TB. For routine TB screening to be effective among HCWs in China, additional research is needed to better understand the low uptake of sputum screening. Alternative laboratory-based screening methods that are more acceptable to HCWs may need to be considered, along with active follow up of presumptive TB cases to ensure they undergo appropriate laboratory-based TB testing.

The study also found that imperfections of the diagnostic tests used in the national TB diagnostic algorithm could hinder its use to conduct successful medical TB surveillance in HCWs. For example, identification of no smear positives in this study may have been partly due to challenges with the AFB sputum test as sensitivity for smear microscopy can range between 20 and 80% based on how samples are collected, stored, transported, and analyzed [17–19]. In contrast, radiography is non-specific for TB diagnosis, which makes diagnosis based on x-ray challenging [20–23]. Similarly, the utility of TST is also debatable, not only for active TB but for latent TB. The validity of TST results using Chinese domestically produced tuberculin have been questioned due to concerns about cross-reactivity with BCG vaccination [4, 7], which is provided to everyone at birth in China. Furthermore, healthcare facilities in China do not routinely perform TST on HCWs because of poor infrastructure for performing TST, high endemic rates of TB transmission, lack of policy on prophylaxis for TB infection, and limited recognition of the contribution of healthcare worker TB surveillance to TB control [7, 9]. A screening algorithm with more accurate testing methods could be needed to make routine medical surveillance for TB worthwhile in China.

The high number of HCWs who met the case definition for clinically-diagnosed pulmonary TB in this study but lacked microbiological testing suggests routine screening for active TB disease among healthcare workers in China could benefit by inclusion of more accurate and better accepted diagnostic tests, like WHO-approved rapid tests (e.g., Xpert® MTB/RIF [Xpert]). Due to their ease of use and rapid turn-around of results, these tests could be more appealing to healthcare workers; expanding use of these WHO-approved rapid tests could thus improve uptake of bacteriological testing. China’s Food and Drug Administration has approved use of Xpert for drug resistance testing among smear positive TB cases [24], but expanding the use of Xpert for TB diagnosis of smear negative TB cases has improved accuracy of TB diagnosis in other high-burden TB settings [25–27]. Although Xpert tests are expensive, modeling suggests that coupling same-day sputum microscopy with Xpert testing could reduce TB incidence and mortality in high-burden settings [28]. Considering the high rate of MDR TB in China, incorporating drug resistance testing into routine medical surveillance for all HCWs would also be beneficial [29].

This evaluation has several limitations. This project was conducted in one autonomous region in China and may not be representative of China as a whole. We relied upon routine diagnostic practices and technologies used at the facility level, so we could not control for the quality of X-rays, sputum collection, or microscopy reading. As a result, quality may have varied substantially between the 28 facilities, which had a wide range of capacity for TB screening and referral. The lack of culture, or other confirmatory tests, within the screening algorithm also made it impossible to assess diagnostic accuracy. In addition, single-step TST tests were conducted which could have led to under-identification of TST positive participants due to the booster effect, thus under-diagnosing participants who met the case definition of clinically-diagnosed pulmonary TB. Conversely, the high prevalence of BCG vaccination in China and the local TST’s potential to cross-react with BCG vaccination could have resulted in an artificially high number of HCWs developing positive TSTs, thus overestimating number of participants who med the case definition of clinically-diagnosed pulmonary TB. Furthermore, as this study relied on the national TB policies and practices already in place for testing presumptive TB patients, facilities only reported on positive smear results, so the number of HCWs who completed AFB sputum testing was unknown; this could have led to underestimation of sputum smear positive TB cases. Similarly, we had to rely on the routine national electronic reporting system to know whether presumptive TB patients were diagnosed with TB after the study period because it was outside the scope of the study to follow HCWs after TB screening. Finally, no information was available on HCWs who chose not to participate in, or were not offered, TB screening, so intrinsic differences between participating and non-participating HCWs are unknown. Additional research is needed to elucidate reasons for HCW participation, or lack of participation, in the TB screening process.

## Conclusions

Although the burden of TB in China has declined substantially since 1990, the country continues to bear a large portion of the world’s incident TB cases, with healthcare workers being at high risk for TB. China currently has no policy on medical surveillance for TB among HCWs. This study suggests that while using the current national TB diagnostic algorithm to screen HCWs for TB in China could be a start to actively find TB in this at-risk population, this framework would require adjustments to increase the ease, accuracy, and acceptance of TB screening in order to achieve optimal medical surveillance for TB in China. To do this, there is a need for stronger approaches to obtain microbiological evaluation on presumptive TB cases, which could include expanded use of Xpert or other WHO-approved rapid tests. Operational research would be necessary to fully elucidate the feasibility, costs, and benefits of adapting a revised TB screening algorithm for routine medical TB surveillance in China.

## Abbreviations

AFB: Acid-fast bacilli
BCG: Bacillus Calmette-Guérin
China CDC: China Center for Disease Control and Prevention
HCWs: Healthcare workers
MDR: Multidrug resistant
TB: Tuberculosis
TST: Tuberculin skin test
US CDC: United States Centers for Disease Control and Prevention
Xpert: Xpert® MTB/RIF
